# Haploinsufficiency of cathepsin D leads to lysosomal dysfunction and promotes cell-to-cell transmission of *α*-synuclein aggregates

**DOI:** 10.1038/cddis.2015.283

**Published:** 2015-10-08

**Authors:** E-J Bae, N Y Yang, C Lee, S Kim, H-J Lee, S-J Lee

**Affiliations:** 1 Neuroscience Research Institute and Department of Medicine, Seoul National University College of Medicine, Seoul 110-799, Korea; 2 Institute of Biomedical Science and Technology, Konkuk University, Seoul 143-701, Korea; 3 Department of Anatomy, School of Medicine, Konkuk University, Seoul 143-701, Korea; 4 ToolGen, Inc., Biotechnology Incubating Center, Seoul National University, Seoul 305-390, Korea

## Abstract

Lysosomal dysfunction has been implicated both pathologically and genetically in neurodegenerative disorders, such as Alzheimer's disease and Parkinson's disease (PD). Lysosomal gene deficiencies cause lysosomal storage disorders, many of which involve neurodegeneration. Heterozygous mutations of some of these genes, such as *GBA1*, are associated with PD. *CTSD* is the gene encoding Cathepsin D (CTSD), a lysosomal protein hydrolase, and homozygous CTSD deficiency results in neuronal ceroid-lipofuscinosis, which is characterized by the early onset, progressive neurodegeneration. CTSD deficiency was also associated with deposition of *α*-synuclein aggregates, the hallmark of PD. However, whether partial deficiency of CTSD has a role in the late onset progressive neurodegenerative disorders, including PD, remains unknown. Here, we generated cell lines harboring heterozygous nonsense mutations in *CTSD* with genomic editing using the zinc finger nucleases. Heterozygous mutation in *CTSD* resulted in partial loss of CTSD activity, leading to reduced lysosomal activity. The *CTSD* mutation also resulted in increased accumulation of intracellular *α*-synuclein aggregates and the secretion of the aggregates. When *α*-synuclein was introduced in the media, internalized *α*-synuclein aggregates accumulated at higher levels in CTSD+/− cells than in the wild-type cells. Consistent with these results, transcellular transmission of *α*-synuclein aggregates was increased in CTSD+/− cells. The increased transmission of *α*-synuclein aggregates sustained during the successive passages of CTSD+/− cells. These results suggest that partial loss of CTSD activity is sufficient to cause a reduction in lysosomal function, which in turn leads to *α*-synuclein aggregation and propagation of the aggregates.

Maintaining protein homeostasis (proteostasis) is crucial in not only maintenance of physiological functions of cells, but survival of cells. Proteostasis is a particularly important issue for the survival of post-mitotic cells, such as neurons, while dividing cells can dilute aged and misfolded proteins during the mitosis process.^1, 2^ For the clearance of protein burden, cells utilize two major protein degradation systems, ubiquitin proteasome system and lysosomal degradation, the latter degrades endosomal and autophagosomal cargos.^3, 4, 5, 6^ Dysregulation of ubiquitin proteasome system and lysosome has been shown to cause protein conformational diseases, including neurodegenerative disorders and metabolic disorders.^7, 8^ Genetic studies have suggested that impairment of lysosomal functions has important roles in the pathogenesis of neurodegenerative diseases. Mutations in *ATP13A2*, *GBA1* and *VPS35* have been associated with PD.^9, 10, 11, 12^ Mutations in progranulin and charged multivesicular body protein 2B (CHMP2B) have been identified as genetic causes of amyotrophic lateral sclerosis and frontotemporal dementia.^13, 14, 15^ Postmortem brain tissues of neurodegenerative diseases have exhibited deposition of endosomal and autophagic vesicles.^16^ Therefore, neurodegenerative proteinopathies might be attributed to lysosomal dysfunction.

Pathological examinations of patient tissues have exhibited that protein aggregates, such as amyloid beta (A*β*), tau and *α*-synuclein aggregates, spread to larger brain regions as disease progresses.^17^ In animal models, intracerebrally injected *α*-synuclein aggregates could spread into larger brain regions both in *α*-synuclein transgenic and non-transgenic mice.^18, 19, 20, 21^ Inoculation of A*β* or tau aggregates into either non-transgenic or transgenic models of AD also exhibited propagation of those aggregates.^22, 23, 24, 25, 26, 27, 28^ Studies have suggested that cell-to-cell transmission of protein aggregates is the underlying mechanism of the pathological propagation.^29, 30^

Mounting evidence have suggested that lysosomal function is important for the clearance of the transferred aggregates in recipient neurons during cell-to-cell aggregate transmission.^31^ This has been extensively studied in cell culture models for *α*-synuclein transmission. Previous studies showed *α*-synculein aggregates can be internalized and transported through the endolysosomal pathway.^32^ Lyososomal dysfunction led to increased accumulation of the internalized *α*-synuclein aggregates, suggesting that the lysosomal activity in recipient cells is critical in the clearance of the transmitted *α*-synuclein aggregates.^32, 33^

Lysosomal storage diseases (LSDs) are caused by defects in the lysosomal degradation process. Mutations in genes encoding lysosomal catabolic enzymes and transporters manifest excessive deposition of the enzyme substrates in various organs.^34^ Though different LSDs show different symptoms, most of LSD patients exhibit neurological symptoms such as mental retardation, motor dysfunction and progressive neurodegeneration, as well as specific pathological changes in the nervous system.^35, 36^ In addition, some of progressive neurodegenerative disorders such as AD, PD and Huntington's disease also show similar pathological features with LSD: accumulations of endosomal and autophagosomal vesicles and undegraded macromolecules, and inflammatory responses in brain.^16^

Gaucher's disease (GD) is the most common LSD, which is inherited in an autosomal recessive manner. Homozygous mutations of *GBA1* gene, encoding *β*-glucocerebrosidase 1 (GCase 1), a lysosomal hydrolase, is responsible for GD.^37^ Evidence has suggested that GD is closely related to PD. Patients with type-1 GD, the most common form of GD, frequently develop parkinsonism.^38^ Heterozygous carriers of *GBA1* mutations are at a higher risk for PD.^39, 40^ It has been shown that about 75% of Lewy bodies, a pathological hallmark of PD, colocalized with GCase 1 in brains of PD and DLB patients with heterozygous *GBA1* mutations.^41^ These results suggest that lysosomal enzyme deficiency is associated with the development of PD.

Cathepsin D (CTSD) is a major lysosomal endopeptidase, which is critical in the degradation of long-lived proteins.^42^ Genetic and clinical studies have shown that the homozygous deficiency of CTSD results in the early onset, progressive neurodegeneration, such as congenital neuronal ceroid-lipofuscinosis.^43^ The heterozygous missense mutations in *CTSD* have been known to cause the early onset motor and visual problems, brain atrophy, and progressive psychomotor symptoms.^44^ However, the effects of CTSD deficiency on the late onset progressive neurodegenerative disorders, including AD and PD, remain unclear. Nevertheless, it has become clear that CTSD activity is crucial in the degradation of pathogenic protein aggregates.^45, 46^

Herein, we generated a cell line with a heterozygous nonsense mutation in *CTSD* and investigated the roles of the CTSD activity in lysosomal function, *α*-synuclein aggregation and transcellular transmission of *α*-synuclein aggregates.

## Results

### Lysosomal dysfunction by reduced CTSD activity

**Figure 1:**
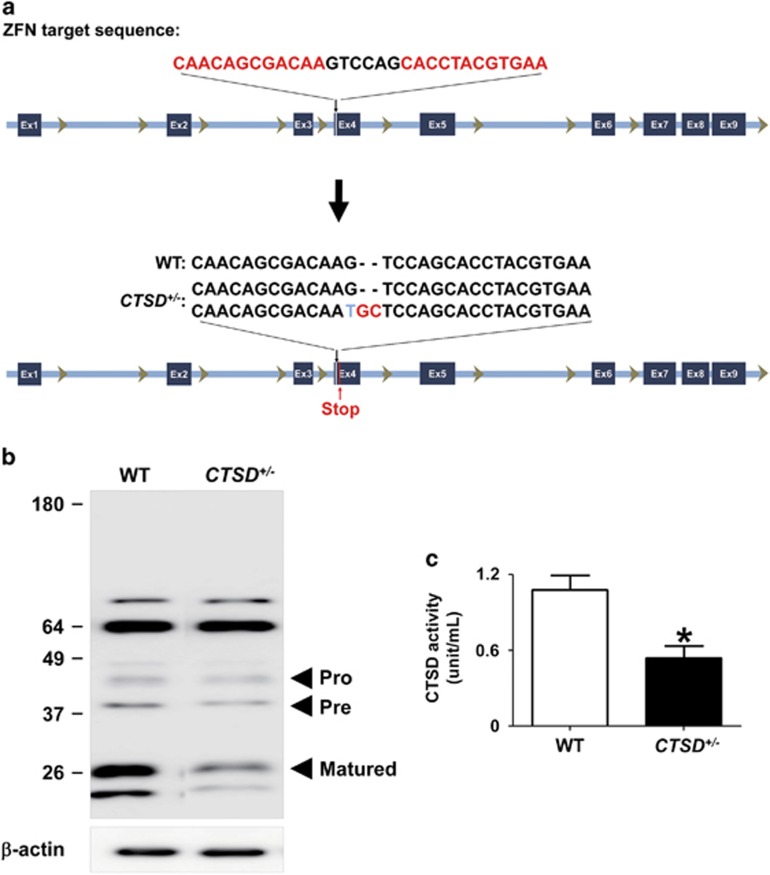
Generation of *CTSD*-deficient SH-SY5Y cell line by using zinc finger nucleases. (**a**) To generate nonsense mutations in the *CTSD* gene, SH-SY5Y human neuroblastoma cells were transfected with zinc finger nucleases targeting exon 4. The nonsense mutations in single copy of *CTSD* exon 4 were confirmed by DNA sequencing. (**b**) Western blot analysis of CTSD in SH-SY5Ycell lysates. (**c**) The significant reduction of intracellular CTSD activity. *n*=4, *P<0.05 by paired, two-tailed Student's *t* test

To investigate the roles of CTSD activity on lysosomal function and *α*-synuclein aggregation, we have generated SH-SY5Y cell lines with nonsense mutations in *CTSD* gene using zinc finger nucleases technology. We designed the zinc finger nucleases that introduce mutations in exon 4 of *CTSD* and obtained six clones with mutations in *CTSD* gene. The presence of nonsense mutations were confirmed by DNA sequencing (Figure 1a). All the clones we selected carried mutations only in a single copy. We were not able to generate a clone with mutations in both copies. This is consistent with the previous transgenic mouse study, in which CTSD-null mice were lethal. Figure 1b showed that the nonsense mutation in *CTSD* (CTSD+/−) resulted in a reduced expression of CTSD (Figure 1b). We measured the intracellular activity of CTSD both in the WT and CTSD+/− cell lines in the presence or absence of pepstatin A, an inhibitor of CTSD. As expected, pepstatin A-sensitive CTSD activity was decreased in CTSD+/− cells (Figure 1c).

**Figure 2:**
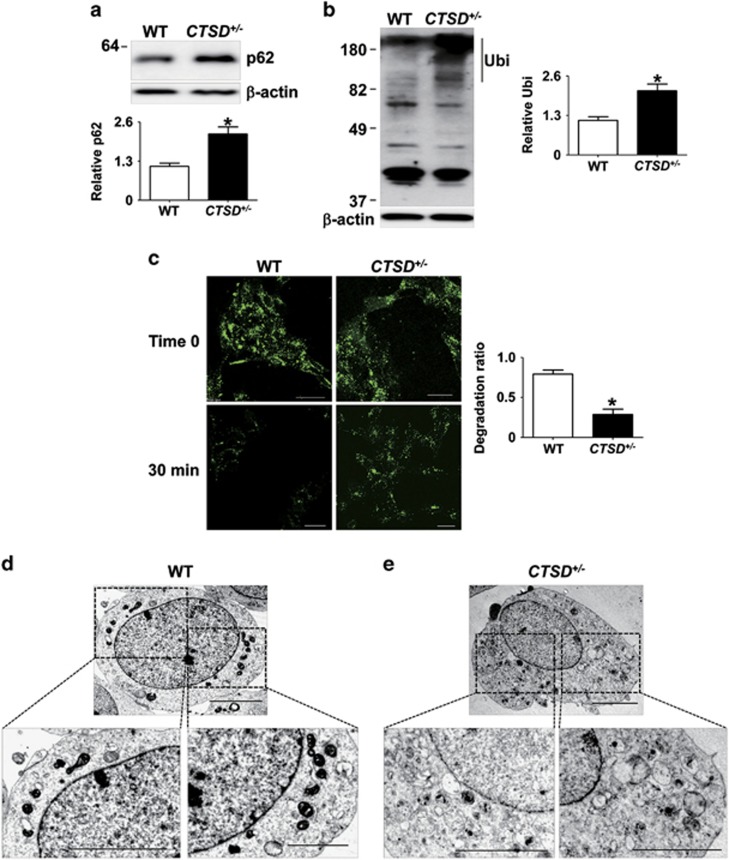
Loss of CTSD activity led to the lysosomal dysfunction. (**a** and **b**) The accumulation of p62 (**a**) and polyubiquitinated proteins (**b**) were analyzed in Triton X-100-insoluble fraction. For quantification of polyubiquitinated proteins, the quantified size range is indicated by the line to the right of the blot. *n*=4, * *P*<0.05 by paired, two-tailed Student's *t* test. (**c**) To test lysosomal degradation rate, degradation rate of internalized dextran–fluorescein isothiocyanate (FITC) was calculated. Scale bars: 20 *μ*m, *n*=4, *P<0.05 by paired, two-tailed Student's *t* test. One hundred cells were analyzed per experiment. (**d** and **e**) Electron microscopy of WT (**d**) and *CTSD*^+/−^ (**e**). The boxed areas in the upper images are magnified in the lower panels. Scale bars: 2 *μ*m

To address the effects of CTSD activity on lysosomal clearance, we measured intracellular accumulation of lysosomal substrates, such as polyubiquitinated proteins and p62, a polyubiquitin binding protein. CTSD+/− cells exhibited higher steady state levels of p62 and polyubiquitinated proteins than WT cells (Figures 2a and b). Next, we examined the lysosomal degradation activity using fluorescence-labeled dextran. CTSD+/− cells exhibited significantly reduced degradation rate of ectopically introduced dextran than WT cells (Figure 2c). We also analyzed the morphological changes in CTSD+/− cells using transmission electron microscopy and found accumulation of autophagic vesicles and damaged cellular organelles (Figures 2d and e). These results suggested that the reduction in CTSD activity caused lysosomal impairment.

### Alterations in *α*-synuclein metabolism by CTSD deficiency

**Figure 3:**
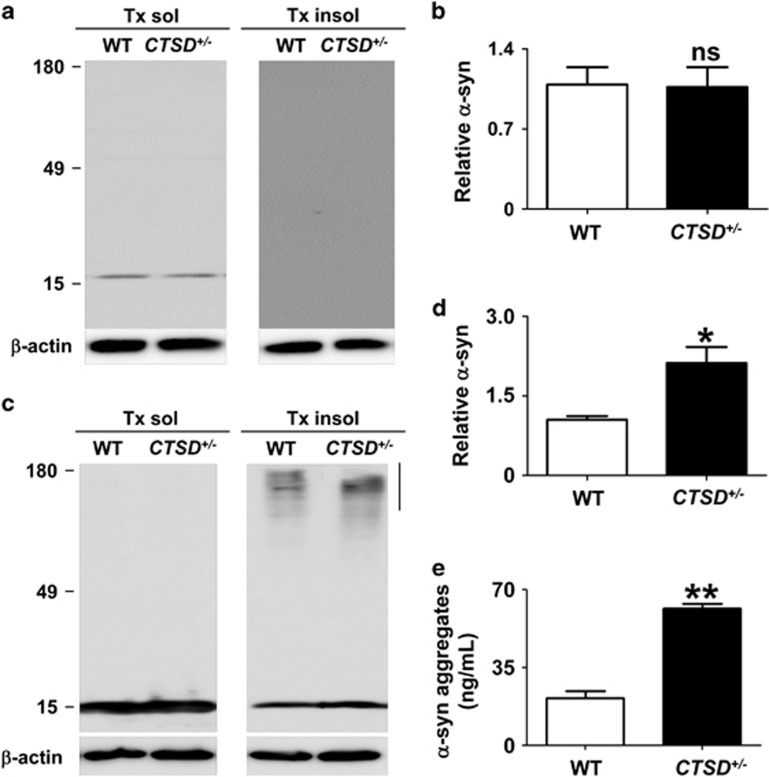
Alterations in accumulation and secretion of *α*-synuclein aggregates by CTSD deficiency. (**a** and **b**) Western blot analysis of *α*-synuclein both in the WT and *CTSD*^*+*/−^ SH-SY5Y cells. After differentiation, the levels of *α*-synuclein was measured in Triton X-100-soluble and -insoluble fraction. Owing to low expression level, *α*-synuclein was not detected in the Triton-insoluble fraction. The amount of *α*-synuclein in the Triton-soluble fraction was analyzed in (**b**). *n*=3. (**c** and **d**) Western blot analysis of *α*-synuclein. Human *α*-synuclein was overexpressed both in the WT and *CTSD*^*+*/−^ SH-SY5Y cells. On day 3 after infection, the levels of *α*-synuclein was measured in Triton X-100-soluble and -insoluble fraction. For quantification of *α*-synuclein aggregates, the quantified size range is indicated by the line to the right of the blot. Loss of CTSD activity increased the accumulation of *α*-synuclein aggregates in the Triton-insoluble fraction (**d**). *n*=3, *P<0.05 by paired, two-tailed Student's *t* test. (**e**) The amount of secreted *α*-synuclein aggregates in cell culture media were measured using *α*-synuclein aggregate-specific ELISA. *n*=4, **P<0.01 by paired, two-tailed Student's *t* test

Previous studies have shown that the lysosomal function is critical for degradation of protein aggregates.^47^ Therefore, we tested whether the loss of CTSD activity might accelerate the accumulation of *α*-synuclein aggregates. First, we analyzed the intracellular amount of the endogenous *α*-synuclein both in WT and CTSD+/− cells. The loss of CTSD activity did not change the levels of the endogenous *α*-synuclein (Figures 3a and b). This result might be because of the low-level expression of the endogenous *α*-synuclein. In other words, even half the normal level of CTSD might be enough to handle the endogenous *α*-synuclein. We then investigated the effects of CTSD mutation after overexpression of *α*-synuclein. Both WT and CTSD+/− cells were infected with the adenoviral vector for human *α*-synuclein, and the intracellular levels of *α*-synuclein were measured. *α*-Synuclein aggregates accumulated at higher levels in the Triton X-100-insoluble fractions of CTSD+/− cells than in those of WT cells, whereas the CTSD deficiency did not alter the levels of monomeric *α*-synuclein (Figures 3c and d).

Previous studies showed that neuronal cells can release small amounts of both monomeric and aggregate forms of *α*-synuclein to extracellular space.^48^ The secretion of *α*-synuclein aggregates was increased under certain stress conditions, such as oxidative stress and proteosomal stress.^49^ We examined alterations in the secretion of *α*-synuclein aggregates in CTSD+/− cells using *α*-synuclein aggregate-specific enzyme-linked immunosorbent assay. The amounts of secreted *α*-synuclein aggregates from CTSD+/− cells was higher than those from WT cells (Figure 3e). These results showed that the reduction in CTSD activity causes an increase in the accumulation and secretion of *α*-synuclein aggregates.

**Figure 4:**
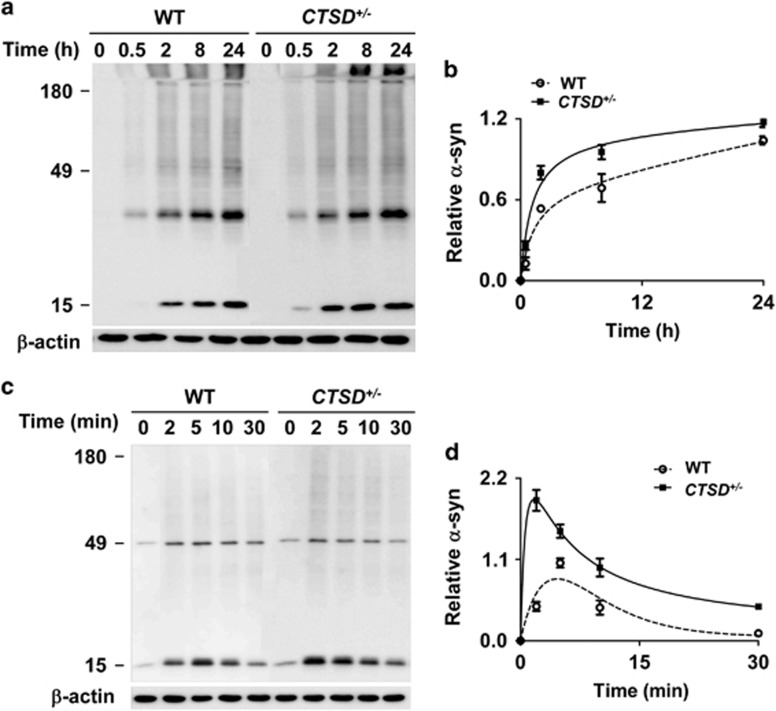
Increased accumulation of exogenous *α*-synuclein aggregates in *CTSD*^*+*/−^ cells. (**a** and **b**) Increased accumulation of *α*-synuclein fibrils in *CTSD*^*+*/−^ cells. Internalized *α*-synuclein fibrils were analyzed by western blotting at the indicated times. The levels of *α*-synuclein aggregates in (**a**) was quantified and presented in (**b**). The relative values to the maximum level of internalized *α*-synuclein in WT cells were presented in the graph in (**b**); *n*=3. (**c** and **d**) Accumulation of cell-derived *α*-synuclein aggregates. Internalized *α*-synuclein aggregates were analyzed by western blotting at the indicated times. The relative levels to the maximum level of internalized *α*-synuclein in WT cells were presented in (**d**); *n*=3

We then examined the role of CTSD in degradation of internalized *α*-synuclein aggregates when the protein was introduced in the culture media. We compared the accumulation kinetics of *α*-synuclein aggregates both in WT cells and CTSD+/− cells. When *α*-synuclein fibrils were administered in the media, the time required for reaching the maximum level was 24 h in WT cells, while the maximum level was reached at 8 h in CTSD+/− cells (Figures 4a and b). When the cell-released *α*-synuclein was introduced to the culture, the time to reach the maximum level was reduced from 5 to 2 min in CTSD+/− cells compared with the WT cells (Figures 4c and d). These results indicate that CTSD+/− cells have reduced ability to degrade the internalized exogenous *α*-synuclein aggregates.

### CTSD deficiency accelerates the transmission of *α*-synuclein aggregates

**Figure 5:**
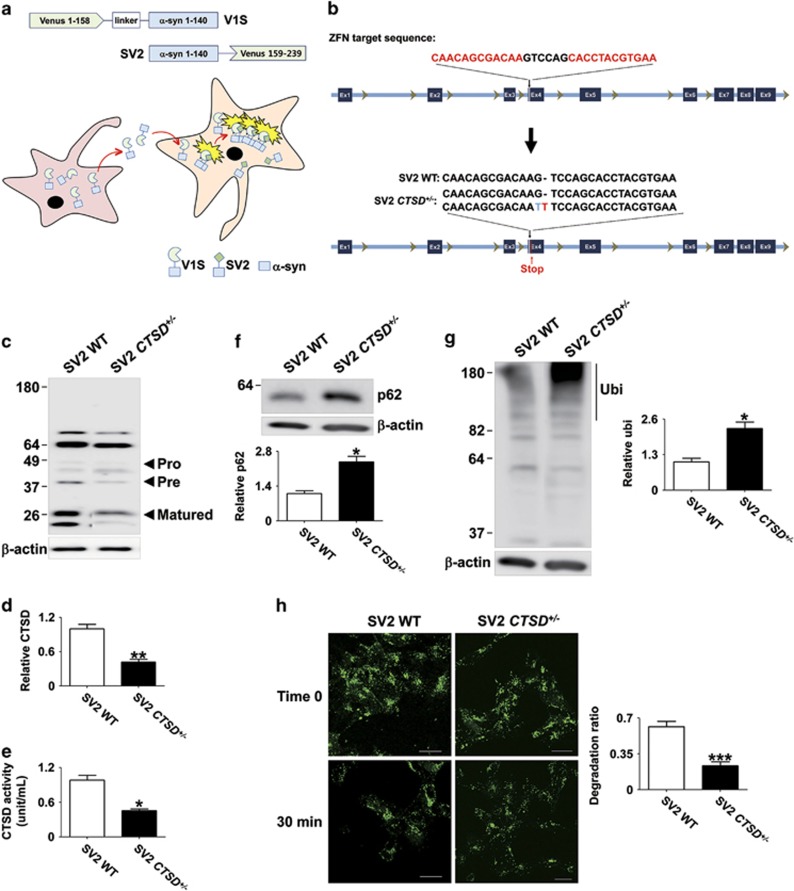
Lysosomal dysfunction in *CTSD*^*+*/−^ SV2 cells. (**a**) Scheme of the dual cell BiFC co-culture system. The system consists of two stable cell lines expressing *α*-synuclein conjugated with either the amino terminus (V1S) or carboxy terminus (SV2) fragment of Venus fluorescence protein. When the cells were co-cultured, cell-to-cell transmission of *α*-synuclein was detected by using BiFC fluorescence resulting from dimerization or oligomerization of the V1S and SV2 fusion proteins. (**b**) Nonsense mutation in single copy of *CTSD* gene in SV2 cells. (**c** and **d**) Western blot analysis showing reduced CTSD expression in SV2 *CTSD+*/− cells. *n*=4, **P<0.01 by paired, two-tailed Student's *t* test. (**e**) Reduction of the intracellular CTSD activity. *n*=4, *P<0.05 by paired, two-tailed Student's *t* test. (**f**) Levels of p62 in the Triton-insoluble fractions. *n*=3, *P<0.05 by paired, two-tailed Student's *t* test. (**g**) Levels of polyubiquitinated proteins. Quantified regions in the blots are indicated by the line on the right. *n*=3, *P<0.05 by paired, two-tailed Student's *t* test. (**h**) Degradation efficiency of imported dextran-fluorescein isothiocyanate (FITC). Scale bars: 20 *μ*m. *n*=4, ***P<0.005 by paired, two-tailed Student's *t* test. One hundred cells were analyzed per experiment

Partial deficiency of CTSD caused both increase in secretion of *α*-synuclein aggregates and reduction in degradation of internalized exogenous *α*-synuclein aggregates. These results strongly implicate increased cell-to-cell transmission of *α*-synuclein aggregates with CTSD defects. To assess the roles of CTSD in transcellular transmission of *α*-synuclein aggregates, we utilized the bimolecular fluorescence complementation co-culture system (BiFC), which has been described previously as a cellular model for the study of cell-to-cell transfer and seeded aggregation of *α*-synuclein.^50^ This model system is composed of two SH-SY5Y cell lines that stably express *α*-synuclein conjugated with either the N-terminal (V1S) or C-terminal (SV2) fragments of the Venus fluorescence protein (Figure 5a). When these stable cell lines are co-cultured, *α*-synuclein proteins were secreted from each cell line and transferred to another stable lines, resulting in co-aggregation between the transferred *α*-synuclein and the intrinsic *α*-synuclein. This co-aggregation leads to reconstitution of Venus fragments and can be monitored by fluorescence emission.

Using the zinc finger nucleases targeting exon 4 in the *CTSD* gene, we have generated the nonsense mutations in the *CTSD* gene in the SV2 cell line (Figure 5b). Like the naive SH-SY5Y cells, we only obtained heterozygous mutant cell lines (SV2 CTSD+/−), and the nonsense mutations in single copy of *CTSD* decreased both the expression and activity of CTSD in SV2 cells (Figures 5c and e). Lysosomal substrates, such as p62 and polyubiquitinated proteins, accumulated at higher levels in SV2 CTSD+/− cells than in SV2 cells (Figures 5f and g). Degradation rates of ectopically introduced dextran were reduced in SV2 CTSD+/− cells compared with SV2 cells (Figure 5h).

**Figure 6:**
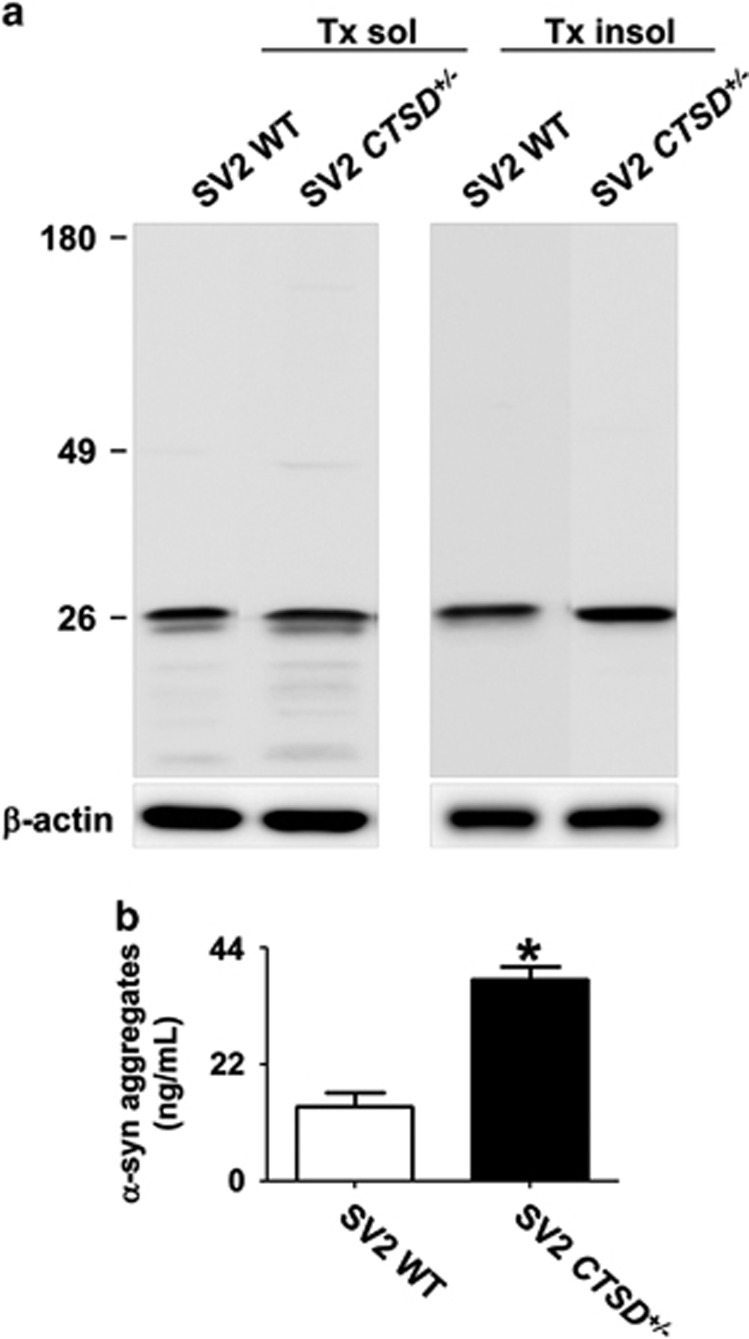
CTSD deficiency increased the secretion of *α*-synuclein aggregates in SV2 cells. (**a**) Western blot analysis of *α*-synuclein. The levels of *α*-synuclein conjugated with C-terminus of Venus fluorescence protein fragment was measured in Triton X-100 soluble and insoluble fraction. (**b**) The amount of secreted *α*-synuclein aggregates in cell culture media were measured using *α*-synuclein aggregate specific enzyme-linked immunosorbent assay. *n*=4, *P<0.05 by paired, two-tailed Student's *t* test

Next, we tested alterations in the accumulation and secretion of *α*-synuclein aggregates in SV2 CTSD+/− cells. Unlike the CTSD+/− cells, the SV2 CTSD+/− did not exhibit increased deposition of *α*-synuclein aggregates compared with the SV2 cells. This is probably due to lower expression levels of *α*-synuclein in SV2 cells than in SH-SY5Y cells infected with the *α*-synuclein adenoviral vector. Nevertheless, secretion of *α*-synuclein aggregates was increased in SV2 CTSD+/− compared with SV2 cells (Figures 6a and b).

**Figure 7:**
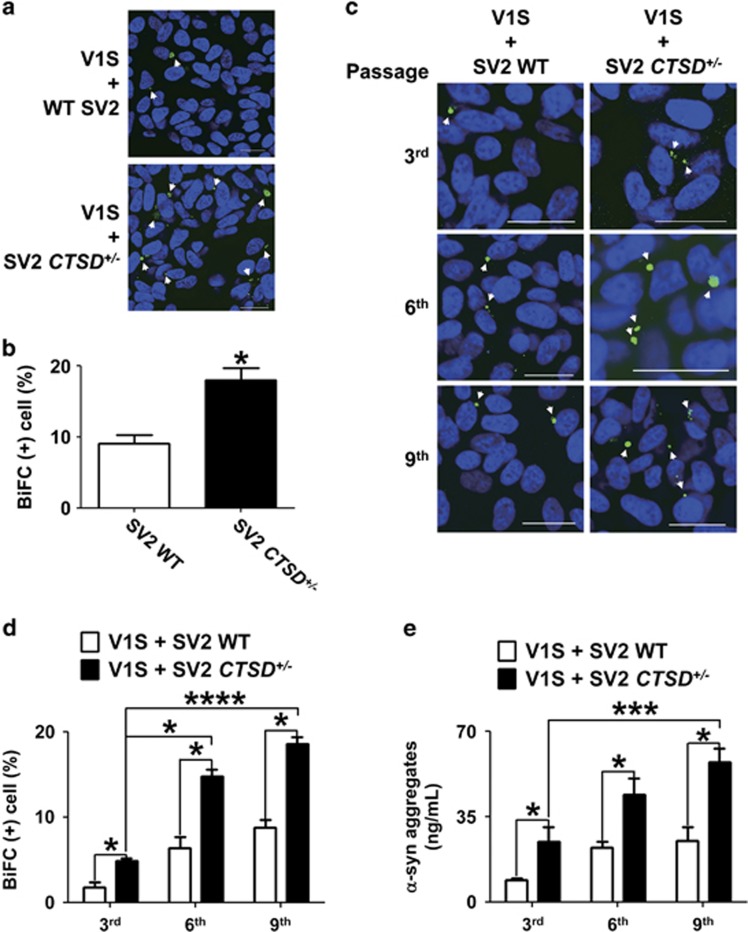
CTSD deficiency increased cell-to-cell transmission of *α*-synuclein aggregates. (**a** and **b**) Increased cell-to-cell transmission of *α*-synuclein aggregates in V1S/SV2 *CTSD*^*+*/−^ co-culture. BiFC-positive aggregates are indicated by arrowheads. Scale bars: 20 *μ*m. The number of BiFC-positive cells in (**a**) was quantified in (**b**); *n*=4, 500 cells per experiment, *P<0.05 by paired, two-tailed Student's *t* test. (**c** and **d**) Increased continuous transcellular transmission of *α*-synuclein aggregates in V1S/SV2 *CTSD*^*+*/−^ co-culture. BiFC-positive aggregates are indicated by arrowheads. Scale bars: 20 *μ*m. The number of BiFC-positive cells in (**c**) was quantified in (**d**); *n*=4, 500 cells per experiment, *P<0.05, ****P<0.001 by paired, two-tailed Student's *t* test. (**e**) *α*-Synuclein aggregate-specific enzyme-linked immunosorbent assay analysis of culture media from the indicated subcultures. *n*=3, *P<0.05, ***P<0.005 by paired, two-tailed Student's *t* test

To directly address alterations in the transmission of *α*-synuclein aggregates, either SV2 or SV2 CTSD+/− cells were co-cultured with V1S cells. Then, we measured BiFC fluorescence, an indicator of co-aggregation between the *α*-synuclein proteins derived from V1S and SV2 cells (Figure 7a).^50^ When V1S cells were co-cultured with SV2 CTSD+/− cells, the number of BiFC fluorescence-positive cells was significantly increased compared with the V1S/SV2 co-culture (Figures 7a and b), indicating that reduction in CTSD expression leads to increased transmission of *α*-synuclein aggregates. In a previous study, we showed that transmission of *α*-synuclein aggregates perpetuates through multiple cells.^50^ To determine the effects of *CTSD* mutation on the perpetual *α*-synuclein transmission, we continuously sub-cultured both V1S/SV2 and V1S/SV2 CTSD+/− co-cultures through several passages. The number of BiFC-positive cells was elevated with increasing passage numbers in both co-cultures, and the percentage of BiFC-positive cells was consistently higher in the V1S/SV2 CTSD+/− co-culture than the V1S/SV2 culture throughout the passages (Figures 7c and d). Consistent with this result, the amounts of secreted *α*-synuclein aggregates were higher in V1S/SV2 CTSD+/− co-culture than in V1S/SV2 co-culture (Figure 7e). Collectively, these results suggest that the reduction in CTSD expression/activity potentiates the transcellular transmission of *α*-synuclein aggregates by accelerating secretion and impairing the clearance of the transferred *α*-synuclein aggregates.

## Discussion

In this study, we showed that experimental haploinsufficiency of CTSD resulted in the reduction of lysosomal functions, impaired *α*-synuclein catabolism, increased *α*-synuclein secretion and decreased degradation of internalized *α*-synuclein, leading to increased cell-to-cell transmission of *α*-synuclein aggregates. The current study suggests that reduction in CTSD activity is sufficient to cause a significant decline of lysosomal functions and accelerate the propagation of Lewy body pathology.

In humans, homozygous mutations of *CTSD* causes neuronal ceroid-lipofuscinosis, an early onset lysosomal storage disease, while heterozygous missense mutations results in early-onset motor and visual disturbances, brain atrophy, and progressive psychomotor problems.^44^ However, the effects of partial decline of CTSD activity on development and progression of late-onset neurodegenerative disease, such as PD, has not been addressed. CTSD is a lysosomal hydrolase, which has important roles in the degradation of long-lived proteins. Ectopic expression of CTSD in cells led to a reduction of *α*-synuclein aggregation and neurodegeneration, whereas neither transduction of cathepsin B nor L was protective against *α*-synuclein aggregates-induced toxicity.^45, 46^ Studies with CTSD-deficient mice showed accumulation of insoluble *α*-synuclein aggregates in the brain, whereas these were absent in wild-type littermates. Cullen *et al.*^46^ also reported the deposition of phosphorylated *α*-synuclein aggregates only in CTSD-mutant sheep, not in the normal subjects. Human postmortem studies with brains from early-onset neuronal ceroid-lipofuscinosis patients showed the accumulation of *α*-synuclein aggregates.^46^ Furthermore, in an *α*-synuclein transgenic fly model, depletion of CTSD accelerated the formation of *α*-synuclein aggregates and the associated pathogenic phenotypes, such as accumulation of numerous vacuoles and disruption of the retina structure.^46^ These results indicate that CTSD is critical for maintaining the levels of *α*-synuclein in check, and therefore, reduced expression of CTSD might increase the risk of synucleinopathies.

In the current study, we examined the effects of partial deficiency of CTSD not only on lysosomal functions and *α*-synuclein catabolism but also on secretion and cell-to-cell transmission of *α*-synuclein aggregates. Our study showed that the reduction in CTSD expression caused a decline in lysosomal function, which in turn led to an increase in the accumulation of intracellular *α*-synuclein aggregates and the secretion of the aggregates. We further demonstrated that partial loss of CTSD resulted in a reduction in clearance of internalized *α*-synuclein. As a combined effect of increased secretion and reduced clearance of *α*-synuclein, CTSD partial deficiency resulted in accelerated cell-to-cell transmission of *α*-synuclein aggregates. The observed effects on cell-to-cell transmission was not limited to a single transmission event, rather continued on to subsequent rounds of transmission. These results provide significant implications in the role of CTSD in metabolism of *α*-synuclein aggregates and in the propagation of *α*-synuclein pathology during the progression of PD and potentially other synucleinopathies.

The mechanism by which lysosomal dysfunction led to the increase in *α*-synuclein secretion is not understood yet. Our earlier studies suggested that *α*-synuclein is secreted through the ER-Golgi-independent unconventional exocytosis.^48, 49^ Exosome-associated secretion and exophagy (exocytosis through the fusion of autophagosome/amphisome with the plasma membrane) have been suggested as the mechanisms of unconventional exocytosis of *α*-synuclein.^51, 52^ Although it has not been experimentally demonstrated, one should also consider lysosomal exocytosis as one of the mechanisms.^53^ The relative contribution and pathophysiological significance of these mechanisms are not clear. It is worth noting that all the exocytosis mechanisms discussed above can be activated by lysosomal dysfunction as alternative pathways to degradation.

Heterozygous mutations in *GBA1*, homozygous mutations of which cause GD, are known as strong genetic risk factors of PD and perhaps DLB.^40^ Individuals carrying *GBA1* mutations are five times more likely to have PD than the normal control. And, compared with the healthy control, an incidence of parkinsonism is 6 to 17 times higher in patients with type 1 GD.^38^ Furthermore, a large multicenter study reported that PD patients with *GBA1* mutations not only had a higher incidence of dementia, but also showed earlier onset and faster progress of disease.^54^ Recently, we have shown that the depletion of *GBA1* resulted in lysosomal defects and accelerated the cell-to-cell transmission of *α*-synuclein aggregates.^50^ These data might explain why carriers of *GBA1* mutations exhibit higher incidence and faster progression of the disease.^50^ In the current study, we show that partial deficiency of CTSD results in the similar phenotypes as GBA1 deficiency, exhibiting decline in lysosomal functions and increased secretion and cell-to-cell transmission of *α*-synuclein aggregates. Given the similarities between *CTSD* and *GBA1* phenotypes in cells, it might be worth investigating the relationships among *CTSD* genotypes, PD incidence, and the rate of disease progression.

Even though most of lysosomal hydrolase activities are redundant, it has been repeatedly shown that a single lysosomal mutation can cause lysosomal dysfunction.^55, 56^ Likewise, only a partial loss of CTSD activity is sufficient to cause lysosomal defects. CTSD heterozygous deficient mice exhibited an increase in LAMP1 levels and were more sensitive to MPTP-induced neurotoxicity than the wild-type controls.^57^ This indicates that a half of the activity of CTSD is not sufficient for maintaining the normal lysosomal functions. These results suggest that at least for some hydrolases, keeping the proper quantities is critical for maintaining the normal lysosomal activity. The CTSD protein levels and activities have indeed been shown to be reduced in PD patients,^58, 59^ although it has not been unanimously agreed upon yet.^39, 58^ We speculate that the proper control of expression of lysosomal hydrolases, perhaps through the activation of transcription factor EB, is important for maintenance of proteostasis, and therefore, for the therapy for neurodegenerative diseases.^60^ Furthermore, genetic and epigenetic changes in genes encoding lysosomal proteins might represent common risks for PD and other neurodegenerative diseases.

In conclusion, haploinsufficiency of CTSD resulted in lysosomal dysfunction and abnormal *α*-synuclein metabolism and secretion, thereby promoting cell-to-cell transmission of *α*-synuclein aggregates. Given the similarities to the *GBA1* studies, our current results suggest that the reduced expression of CTSD leads to accelerated disease progression in Lewy body diseases. Our study shows that lysosomal functions could be sensitive to quantities of individual hydrolases. We propose a hypothesis that quantitative changes in certain lysosomal gene expression is the determining factor for development and/or progression rate of PD and other synucleinopathies.

## Materials and Methods

### Materials

The following antibodies were used in this study: CTSD monoclonal antibody CTD-19 (ab6313; Abcam, Cambridge, MA, USA; 1 : 1000 dilution), *β*-actin monoclonal antibody AC-15 (A5441; Sigma-Aldrich, St. Louis, MO, USA; 1 : 10 000 dilution), p62 monoclonal antibody p62 LCK ligand (610833; BD Transduction Laboratories, Swampscott, MA, USA; 1 : 1000 dilution), ubiquitin polyclonal antibodies (Dako, Glostrup, Denmark, and Chemicon, Temecula, CA, USA; 1 : 1000 dilution), *α*-synuclein monoclonal antibody (610787; BD Biosciences, San Diego, CA, USA; 1 : 1500 dilution), *α*-synuclein monoclonal antibody Ab274 (1 : 1500 dilution), *α*-synuclein monoclonal antibody Ab62 (1 : 1000 dilution), HRP-conjugated goat anti-mouse IgG (H+L) (172-1011; Bio-Rad Laboratories, Hercules, CA, USA; 1 : 3000 dilution) and HRP-conjugated goat anti-rabbit IgG (H+L) (Bio-Rad Laboratories; 1 : 3,000 dilution).

Fluorescein-conjugated dextran (10 000 molecular weight; D-1821), TO-PRO-3 iodide (T3605) and LysoTracker Red DND-99 (L-7528) were purchased from Invitrogen (Carlsbad, CA, USA).

### Generation of *CTSD* knockout cell lines

Plasmids encoding zinc finger nuclease and a magnetic reporter (ToolGen, Seoul, Korea) was transfected to SH-SY5Y cells (CRL-2266; ATCC, Manassas, VA, USA) by using electroporation. After incubation for 48 h, transfected cells were enriched by magnetic separation. Briefly, cells were mixed with magnetic bead-conjugated antibody against H-2K^k^ (MACSelect Kk microbeads; Miltenyi Biotech, Gladbach, Germany), and the mixture was applied to a MACS LS column (Miltenyi Biotech). Single cells were isolated from the eluates and maintained until the clonal colonies were picked from the culture dish. Nonsense mutations in the *CTSD* gene were confirmed by DNA sequencing. Six clones with non-sense mutations in exon 4 were generated. Among these clones, two clones were further analyzed.

### Cell culture

SH-SY5Y human neuroblastoma cell lines were cultured as described previously.^50^ Cells were split every 2 days at 37 °C in humidified air with 5% CO_2_ in Dulbecco's modified eagle's medium (SH30243.01, HyClone, Logan, UT, USA) containing 10% fetal bovine serum (SH30396.03, HyClone), 100 units/ml penicillin and 100 units/ml streptomycin (15140-122, Gibco, Grand Island, NY, USA). To differentiate SH-SY5Y cells, cells were cultured in the presence of 50 *μ*M all*-trans* retinoic acid (R2625, Sigma-Aldrich). For overexpression of human *α*-synuclein, differentiated SH-SY5Y cells were infected with a recombinant adenoviral vector (serotype Ad5, CMV promoter) containing human *α*-synuclein cDNA at a multiplicity of infection of 33.3.

For co-culture, V1S and SV2 (or SV2 *CTSD*^*+*/−^) stable cells (180 000 cells each) were mixed in a coverslip and cultured for 3 days. In order to investigate the continuous transmission of *α*-synuclein, the mixture of V1S and SV2 (or SV2 *CTSD*^*+*/−^) cells was sub-cultured every 2 days (48 h).

### Preparation of conditioned medium

Differentiated SH-SY5Y cells were infected with a recombinant adenoviral vector as described above. On day 2 post infection, cells were washed three times with fresh Dulbecco's modified eagle's medium and cultured in serum-free Dulbecco's modified eagle's medium. After 18-h incubation at 37 °C, conditioned medium was collected and centrifuged at 4 °C at 250 × *g* for 10 min, followed by centrifugation at 10 000 × *g* for 10 min at 4 °C to remove cell debris. The conditioned medium was concentrated using an Amicon 10 K MWCO filters (Millipore, Tullagreen, Ireland).

### Preparation of cell extracts

After washing twice with ice-cold PBS, cells were lysed in extraction buffer (1% Triton X-100, 1% (v/v) protease inhibitor cocktail (Sigma, St. Louis, MO, USA) in PBS). Cell lysates were incubated on ice for 10 min and centrifuged at 16 000 × *g* for 10 min. The Triton X-100 insoluble fraction was resuspended in 1 × Laemmli sample buffer and sonicated briefly.

### Western blotting

Western blotting was performed as previously described.^50^ Images were obtained using the FUJIFILM Luminescent Image Analyzer LAS-3000 and analyzed with Multi Gauge (v3.0) software (FUJIFILM, Tokyo, Japan).

### CTSD activity assay

Cellular CTSD activity was determined using a CTSD assay kit purchased from Sigma (CS0500). The assay was carried out as described by the manufacturer's protocol. Briefly, CTSD activity was determined using an internally quenched fluorescent substrate, MCA-Gly-Lys-Pro-Ile-Leu-Phe-Phe-Arg-Leu-Lys(DNP)-D-Arg-NH2 trifluoroacetate salt. Cells were lysed in lysis buffer (0.5% CHAPS in PBS). Ten micrograms of cell lysates were mixed with internally quenched fluorescent substrate either in the presence or absence of 0.2 mg/ml pepstatin A solution, a CTSD inhibitor. After incubation at 37 °C for 10 min, the fluorescence was measured at 10-min intervals (excitation at 328 nm, emission at 393 nm). The activity unit was calculated by using standard reaction curve of recombinant CTSD enzyme.

### Immunofluorescence staining

The procedure for immunofluorescence staining was performed as previously described.^50^ Briefly, cells grown on poly-L-Lysine-coated coverslips were fixed in 4% paraformaldehyde in PBS and permeabilized in 0.1% Triton X-100 in PBS. After incubation with the blocking solution (5% bovine serum albumin/3% goat serum in PBS), cells were incubated with primary antibodies diluted in blocking solution. After washing with ice-cold PBS, cells were incubated with fluorescent dye-conjugated secondary antibodies diluted in blocking solution. Nuclei were stained with TOPRO-3 iodide (Invitrogen). Cells were mounted onto slide glasses in the presence of Prolong Gold Antifade Reagent (Invitrogen). Images were obtained by using Olympus FV1000 confocal laser scanning microscopy (Olympus, Tokyo, Japan).

### Dextran degradation assay

To determine the degradation ratio of ectopically transduced dextran, cells were incubated with 20 *μ*g/ml of fluorescein isothiocyanate-labeled dextran (Invitrogen) for 2 h. After washing with Dulbecco's modified eagle's medium, cells were incubated with fresh growth medium for 30 min. Cells were fixed with a 4% PFA solution. The fluorescence intensity was measured using Olympus FV1000 software. The extent of degradation of internalized dextran–fluorescein isothiocyanate was calculated using the following equation: (*F*_time0_ − *F*_time30_)/*F*_time0_. *F*_time0_ and *F*_time30_ are the integrated fluorescence intensities at 0 and 30 min, respectively.

### Electron microscopy

After fixation with Karnovsky's fixative solution (2% glutaraldehyde, 2% paraformaldehyde and 0.5% CaCl_2_), cells were immersed in 1% osmium tetraoxide for 1.5 h. After dehydrating with 50, 60, 70, 80, 90, 95 and 100% absolute ethanol, cells were infiltrated with propylene oxide and EPON mixture (EPON 812, MNA, DDSA, DMP30) for 10 min before being embedded in EPON mixture. After embedding, cells were sectioned with an EM UC-7 Ultramicrotome (Leica Microsystems, Vienna, Austria) and stained with 6% uranyl acetate and lead citrate. Grids were observed using a transmission electron microscope (JEM-1011, JEOL, Tokyo, Japan) and analyzed using MegaView III software (Soft Imaging System, Münster, Germany).

### Enzyme-linked immunosorbent assay

Enzyme-linked immunosorbent assay was performed as previously described.^61^ Briefly, 96-well enzyme-linked immunosorbent assay plates (Nalge Nunc International, Rochester, NY, USA) were coated with 1 *μ*g/ml of capture antibody (Ab62) in 50 mM carbonate buffer (pH 9.6) at 4 °C overnight. After washing with PBS with 0.05% Tween 20 (PBST), SuperBlock T20 (PBS) Blocking Buffer (Thermo Scientific, Rockford, IL, USA) was added to each well. After incubation for 1 h at room temperature with shaking, plates were washed five times in PBST. Samples and standards were incubated at room temperature for 2.5 h with shaking. After washing with PBST, 1 *μ*g/ml of biotinylated Ab62 in blocking buffer was added to each well. After incubation for 1.5 h at room temperature, the plates were washed with PBST. Avidin-conjugated peroxidase (ExtrAvidin, Sigma) was added to each well. After incubation for 1 h at room temperature, plates were washed with PBST. One hundred microliters of 3,3′,5,5′-tetramethylbenzidine solution (Sigma) were added to each well and incubated for 15 min with shaking. To stop the reaction, 50 *μ*l of 2 N H_2_SO_4_ was added to each well. The absorbance was measured at 450 nm.

### Statistical analysis

Values shown in the figures are means±S.E.M. To analyze the statistical significance, *P* values were calculated by means of paired, two-tailed Student's *t* tests by using InStat version 3.05 software (GraphPad Software, San Diego, CA, USA).

## Abbreviations

AD: Alzheimer's disease
BiFC: Bimolecular fluorescence complementation
CHMP2B: Charged multivesicular body protein 2B
CTSD: Cathepsin D
GCase 1: Glucocerebrosidase 1
GD: Gaucher's disease
LSD: Lysosomal storage disease
PD: Parkinson's disease
